# Two Cases of Post-partum Hæmorrhage Treated by the Injection of Perchloride of Iron

**Published:** 1875-03-01

**Authors:** W. P. Swain


### TWO CASES OF POST-PARTUM HÆMORRHAGE TREATED BY THE INJECTION OF PERCHLORIDE OF IRON.

BY. W. P. SWAIN, F.R.C.S.

*From the Obstetrical Journal of Great Britain and Ireland, Jan.*, 1875.

WITHOUT wishing to fan into a flame the dying embers of controversy on the subject of the injection of perchloride of iron in post-partum hæmorrhage, I desire to put on record the two following cases which have occurred in my practice during the last six months:

CASE I was that of an exceedingly fine, handsome lady, a primipara. Her pregnancy had been uninterruptedly good. Labor commenced on the evening of January 21st. The presentation was normal, the pains good, and a fine male child was born about half-past seven on the morning of the 22d. I gave a small quantity of chloroform at the last. Immediately after the delivery of the child, I found a second head presenting, and, as the pains were not very expulsive, I put on the forceps and delivered a second male child in a few moments. I then removed the placenta, which was single and of enormous size. Considerable hæmorrhage occurred at the moment the placenta was expelled, but the womb contracted firmly, and a binder was put on. Within a few minutes, however, I found the uterus largely expanded, and, on pressure, a huge clot was expelled. I immediately introduced my left hand into the uterus, making pressure externally with the right, but was unable to produce any uterine contraction. During the whole time there was a constant and excessive flow of blood from the vagina, and the patient became collapsed. I administered brandy in large quantities and ergot, and the moment ice could be obtained I placed a large lump in the cavity of the womb, and a bag of ice on the abdomen. All was, however, of no avail, and my hopes of saving the patient were at zero, In the meantime I had obtained further professional assistance, and, with the concurrence of my father and Mr. Whipple, I injected perchloride of iron into the uterus, in the manner advised by Dr. Barnes. From the moment of the injection all flow of blood ceased, although the uterus remained for some time flaccid. The lady made an excellent recovery, and, with the exception of a little lymphatic tenderness in the right thigh, complained of nothing during her convalescence. I should mention that on the second day, and on every succeeding day for some time the vagina was thoroughly well syringed out with Condy's fluid and water.

CASE II.—I was asked by Surgeon-Major Ferguson to see at the Woman's Hospital, on July 9th, a soldier's wife in her third pregnancy. She had a contracted pelvis in its antero-posterior diameter. In her first confinement she remained a hundred and eight hours in labor; in her second, eighty-seven hours. No instrumental assistance was afforded on either occasion, and the children were still-born. This time she had been in labor sixty hours, and I found the head well down on the pelvis, but tightly fixed there. I suggested the application of the forceps, and in a very short time, after the use of considerable traction, the child was born alive, but lived only for half an hour. The placenta was removed at once without difficulty, and the uterus contracted for a short time, but in a few moments tremendous hæmorrhage set in, the blood being projected from the vagina on the floor some distance from the bed. I immediately passed my left hand into the uterus, cleared out its contents, and endeavored to secure contraction, whilst cold water was poured on to the abdomen from a height, and brandy freely administered. This, however, did not correct the bleeding, although it somewhat lessened in quantity. Feeling that another gush of hæmorrhage might be fatal, I injected the perchloride, all the materials being fortunately at hand. The uterus at once contracted, all hæmorrhage ceased, and I hear from Dr. Ferguson that the woman has made an admirable recovery.

I think I may fairly claim two lives for the perchloride treatment. In the first case, all the usual resources, excepting galvanism, were tried in vain; the lady lay dying before our eyes, and there can be no doubt that any further hæmorrhage must have turned the balance against her. In the second case, so rapid and so large was the loss of blood, that nothing short of *immediate* arrest could have saved the patient; and this the perchloride certainly effected.

---

Dr. Richardson on the Effect of Blood-letting in Eclampsia.—On May 29, 1873, Mr. Burton, of Richmond-Terrace, West Brompton, summoned me to see a lady whose symptoms were nearly the same as in the case described above (eclampsia). In this lady the most marked peculiarity of the labor, so Mr. Burton told me, was the excessive discharge of amniotic fluid. After delivery she became comatose, insensible, and vehemently convulsed. Her face was congested, her veins tense, her pupils fixed, and her temperature high, 103° Fahr. Before my arrival the patient had been bled freely from the arm, and the more alarming symptoms had subsided; but the jugular veins were still tense, the temperature was 102° Fahr., and the unconsciousness continued with an occasional convulsion. Confirming fully the treatment that had been pursued, I recommended the abstraction of still more blood by leeches, together with continued application of a collar of ice around the neck. I learned afterwards from Mr. Burton that the amendment in the symptoms continued, and that recovery took place without any interruption. Mr. Burton was so good as to take me to see this patient a few months ago. I found her quite well, and I could gather nothing from her that could lead to the remotest suspicion of her having suffered one single harm from the remedy which, I am morally sure, saved her life.

Note.—The phenomena of coma and convulsion after labor, in cases such as are here described, are, I believe, phenomena of uremia. The symptoms are identical with those of uremic coma, and the cause is not far to seek. The kidney, subjected to intense pressure during the latter term of pregnancy, is suddenly relieved by the escape of the excessive amount of amniotic fluid and birth of the child. Thereupon into the half-paralyzed vessels of the kidney there is an influx of blood, a temporary but effective congestion, and a state of things analogous to that which occurs when the nervous supply of the kidney is divided. The effect of the abstraction of blood in this condition is to afford immediate relief to the congestion of the vessels, to enable the kidney to resume function, and to let the order of the economy proceed.—*Obstetrical Journal.*

---

Copaiba in Dropsy.—Dr. Falconer (*Canada Lancet*, Dec., 1874) feels confident that very few practitioners have given this drug a fair trial in ascites. Passing by cases of cirrhosis and chronic peritonitis, with their accompanying effusions, in which, after reaccumulation after tapping, he has succeeded in not only diminishing but in completely removing the fluid by the use of copaiba, he refers to a case of ovarian dropsy, which he treated successfully by the drug. Copaiba is the diuretic, par excellence, and never fails in removing the serum through the kidneys, except in chronic albuminuria. It obviates the use of elaterium or gamboge, tapping or ovariotomy, in many cases.

CHLOROFORM IN CONGESTIVE CHILLS.—Dr. R. K. Hinton writes to the *Philadelphia Medical and Surgical Reporter*, as follows :

I have been in the habit of using chloroform in the cold stage of congestive chills, and also ordinary intermittents, for several years past, and have never found anything that would bring on reaction more speedily. The following case which I transcribe from my case-book, will show its effects :

On the fifth of July, 1874, I was called in haste to see Patrick O'Connor. The messenger informed me that he was laboring under his third sinking chill. On my arrival, I found my patient pulseless at the wrist; feet and legs cold; constant nausea and vomiting; had had two attacks of syncope; in short, I supposed my patient would never react. Sinapisms were applied to the stomach and epigastric region, and the extremities rubbed with liquid ammonia. Hot baths could not be used, owing to syncope coming on immediately on attempting to raise him up. I immediately put him on six drop doses of chloroform in sweetened water, to be repeated every fifteen minutes. After taking the second dose, he was much better; pulse returning at the wrist; nausea and vomiting entirely stopped. In one hour from this time, reaction was entirely established; the fever soon passed off; and after the free use of quinine, he had no return of the chills.

*Remarks.*—As I before said, I have been in the habit of using this remedy for several years past. The first case was a negro boy, who was laboring under his second chill. He had obstinate vomiting; the whole array of remedies was brought to bear in this case, but to no purpose. Chloroform was substituted, and in a comparatively short time reaction was fully established.

It appears to fulfill the following indications: 1. It allays nausea and vomiting. 2. It allays the pain in the stomach. 3. It equalizes the circulation. 4. Reaction is never excessive after its use. I have used chloroform in the hot stages of intermittent, and with the happiest results. It appears to allay nervous excitement, and by so doing, quiets the heart's action. I have also used it in the treatment of pneumonia, in from five to ten drop doses every two hours. It allays the cough and all nervous excitement, and appears to cool down the fever.

---

THE ANTIPHLOGISTIC ACTION OF CARBOLIC ACID.—Professor Hueter, of Greifswald, in a report to the Third Congress of the German Surgical Society, read a paper on the antiphlogistic action of parenchymatous injections of carbolic acid. He has used this treatment with success in white swelling of the joints, in subacute inflammation of the lymphatic glands, in acute phlegmonous inflammations, and in traumatic erysipelas. The injection fluid consists of a two per cent. watery solution of carbolic acid. This strength may, however, be increased. Two grammes may be injected at one time.

Hueter has also employed this treatment in commencing inflammation of the bones, before suppuration has begun. He recommends the injection of one or two drachms of the mixture into the medullary cavity, the softened cortical substance presenting no material resistance to the point of the needle. This same solution he has employed for the radical cure of hydrocele, injecting about seven grammes into the tunica vaginalis. Here, as in the other cases, the carbolic acid injections show their anæsthetic action, very little pain being caused by the process and no pain following it. In applying this method of treatment to tumors he had no success in the case of lipomas and sarcomata. He succeeded in reducing the size of soft fibromata and myxomata; scirrhous nodules became smaller and painless, and epithelial ulcers were changed into healthy, granulating surfaces.—*Boston Medical and Surgical Journal.*

THE PHYSIOLOGY OF VOMITING, AND THE ACTION OF DRUGS UPON IT.—Dr. T. Lauder Brunton, the present editior of *The Practitioner*, in a highly interesting article on the above subject, submits the following conclusions:

(1.) Vomiting consists in two factors, viz., (1) the simultaneous compression of the stomach by the abdominal muscles and diaphragm; and (2) the opening of the cardiac orifice by the contraction of the longitudinal fibres of the œsophagus.

(2.) When innervation is disturbed, these two factors do not occur together, and thus retching may occur without vomiting.

(3.) The movements of vomiting are correlated by a nervous centre in the medulla oblongata, from which impulses are sent down through various motor nerves to the muscular structures engaged in the act.

(4.) This nervous centre is probably closely connected with the respiratory centre, but is not identical with it.

(5.) It is usually set in action reflexly by irritation of the pharyngeal, gastric, hepatic, enteric, renal, uterine, ovarian, and possibly also by the pulmonary and vesical nerves which come from the periphery towards it. It may also be excited by impressions sent down to it from the brain.

(6.) Vomiting may be arrested in two ways, either by removing the irritant which is exciting the vomiting centre, or by lessening the excitability itself, so that the centre no longer responds to the impressions made on it from without.

(7.) Emetics may be divided into two classes: those which act only on the stomach, and those which act on the vomiting centre also.

(8.) Tartar emetic probably acts in both ways. Tolerance of it is probably due to want of hydrochloric acid in the stomach.

(9.) Emetics may be used to evacuate the stomach and duodenum. They thus remove irritating matters, poisons generated in the stomach by putrefaction, bile, and metals or fever poisons circulating in the entero-hepatic circulation.

(10.) They may be also used to empty the bronchi and gall-bladder, or to cut short epileptic, and to prevent ague fits.—*The Practitioner*, December, 1875.—*N. Y. Med. Record.*

---

INFLUENCE OF NUTRITIVE CHANGES AND EXTERNAL CIRCUMSTANCES IN THE PARENT UPON THE DEVELOPMENT OF SCROFULOUS CHILDREN.—In a recent address by Dr. Evory Kenedy, before the Dublin Obstetrical Society, the proposition was put forward that scrofula is affected by the surroundings and circumstances which modify the human organism, his views being founded upon the fact that "all organizations are not merely variable, but varying." The following case was mentioned as an interesting illustration of the manner in which external circumstances may lead to the production of those modifications of the human organism, which we collectively term "scrofula," and of a return to the normal condition when the circumstances which had produced the changes had passed away. A peasant, whose family was liable to scrofulous modification, as seen markedly in his sister and her family, married a woman free from any taint of scrofula, and had two children, who presented none of the characteristics known as scrofula. After their birth, the father had an attack of rheumatic fever, which left him with an injured heart, and consequently in straitened circumstances from his crippled condition as a bread winner. During this period of nipping poverty, two other children were born, whose clumsy fingers, thick joints, tumid alæ nasi and upper lips, together with a strong tendency to glandular enlargements, mark the scrofulous diathesis. After this time, the mother had an annuity left her, which once more placed them in comparative plenty. Two more children were born, approaching the healthy type of the two eldest, and comparatively free from the characteristics of the middle pair. When the family are gathered together, the history of the married life of the parents can be read in the physiques of their offspring.—*Br. Med. Jour.*—*N. Y. Med. Record.*

———

ABSENCE OF THE FŒTAL PULSE DURING EXTRACTION BY THE FEET.—Professor Dohen, of Marburg, reports* two cases where, during an extraction by the feet, the fœtal pulse ceased for a considerable length of time, and yet with no fatal result to the child. In both cases the pelvis was contracted, and the pulse was distinctly felt to cease beating as the aftercoming head descended into the pelvis. In the first case the cardiac pulsations were suspended for at least two minutes, and in the second case for more than three minutes. In the first case the baby, a girl, was reanimated in a warm bath after about a quarter of an hour's effort; but in the second case the measures to resuscitate the infant, a boy, were continued for half an hour before the frequency of the inspiratory and pulse movements became normal.

Professor Dohen considers that the arrest of the heart in these two cases was due to the compression of the brain and irritation of the pneumogastric. In cases where the arrest of the heart's beat is due to asphyxia, there is but little hope of saving the child's life; but where the action of the pulse is slackened or arrested by compression of the brain, a rapid extraction of the head will usually be sufficient to save the life of the infant.

These views are strictly in accordance with the theory of Leyden, † who has shown that the compression of the brain which slackens the heart's beats may also completely arrest them, and that this action is produced by the help of the pneumogastric.—*Boston Med. and Surg. Jour.*

* *Obstetrical Journal of Great Britain and Ireland*, January, 1875, from *Archives de Tocologie*, October, 1874.

† *Virchow's Archiv.*, xxxvii., 4.

RHEUMATISM.—Dr. T. S. Dowse, in a paper read before the Norwich meeting of the British Medical Association, and published in full in the *Journal*, says that in the treatment of rheumatic fever the first thing to do is to eliminate the acid products of the diseased state; and the next, to relieve pain. To bring this about, he has for the last three years been in the habit of packing most of his cases in a wet blanket, and then rolling them up in dry blankets, so as to promote profuse sweating, and also increase the temperature. Finding this rough method gave good results, he at once adopted a systematic mode of procedure, which he thus describes: The bed is covered with india-rubber sheeting; over this is laid a blanket which has been wrung out of hot water. The patient is then enveloped in the blanket, and covered with six folds of dry blanketing. By this the temperature is raised, and profuse sweating results; the former, if need be, is assisted by the administration of brandy in half-ounce or ounce doses every hour, and the latter by giving freely warm milk and water. If the temperature exceed 102° F., then the stimulant is unnecessary.

Dr. Dowse continues the treatment for three days. He finds that after the third pack the pain has completely subsided, and the sour taste usually disappears.

We may remark that this method of treatment is by no means new.—*The Doctor.*

PRACTICE FOR SALE.

A Physician's practice, good will, etc., at one of the famous Springs, South. Price, $20,000 cash, or good secured paper. The practice now amounting to upwards of $40,000 actual cash receipts yearly, and increasing. The Physician who wishes to sell, has amassed a competency, and wishes to retire. For full particulars, please address, L. A. GILBERT,
206 La Salle Street,
Chicago, Ill.

